# Investigation of salt tolerance in cotton germplasm by analyzing agro-physiological traits and ERF genes expression

**DOI:** 10.1038/s41598-024-60778-0

**Published:** 2024-05-23

**Authors:** Muhammad Mubashar Zafar, Abdul Razzaq, Waqas Shafqat Chattha, Arfan Ali, Aqsa Parvaiz, Javaria Amin, Huma Saleem, Abbas Shoukat, Khalid M. Elhindi, Amir Shakeel, Sezai Ercisli, Fei Qiao, Xuefei Jiang

**Affiliations:** 1 Sanya Institute of Breeding and Multiplication/School of Tropical Agriculture and Forestry, Hainan University, Sanya, China; 2 Department of Plant Breeding and Genetics, University of Agriculture Faisalabad, Faisalabad, Pakistan; 3 Institute of Molecular Biology and Biotechnology, The University of Lahore, Lahore, Pakistan; 4 FB Genetics, Four Brothers Group, Lahore, Pakistan; 5 Department of Biochemistry and Biotechnology, The Women University Multan, Multan, Pakistan; 6 Department of Agricultural Biotechnology, Erciyes Üniversitesi, Kayseri, Turkey; 7 Institute of Soil and Environmental Sciences, University of Agriculture Faisalabad, Faisalabad, Punjab Pakistan; 8 Plant Production Department, College of Food and Agriculture Sciences, King Saud University, P.O. Box 2460, 11451 Riyadh, Saudi Arabia; 9 Department of Horticulture, Faculty of Agriculture, Ataturk University, Erzurum, Turkey

**Keywords:** Antioxidants, Natural variation, Sustainable agriculture, Salt tolerance, Upland cotton, Genetics, Plant sciences, Climate sciences

## Abstract

The development of genotypes that can tolerate high levels of salt is crucial for the efficient use of salt-affected land and for enhancing crop productivity worldwide. Therefore, incorporating salinity tolerance is a critical trait that crops must possess. Salt resistance is a complex character, controlled by multiple genes both physiologically and genetically. To examine the genetic foundation of salt tolerance, we assessed 16 F1 hybrids and their eight parental lines under normal and salt stress (15 dS/m) conditions. Under salt stress conditions significant reduction was observed for plant height (PH), bolls/plant (NBP), boll weight (BW), seed cotton yield (SCY), lint% (LP), fiber length (FL), fiber strength (FS), potassium to sodium ratio (K^+^/Na^+^), potassium contents (K^+^), total soluble proteins (TSP), carotenoids (Car) and chlorophyll contents. Furthermore, the mean values for hydrogen peroxide (H_2_O_2_), sodium contents (Na^+^), catalase (CAT), superoxide dismutase (SOD), peroxidase (POD), and fiber fineness (FF) were increased under salt stress. Moderate to high heritability and genetic advancement was observed for NBP, BW, LP, SCY, K^+^/Na^+^, SOD, CAT, POD, Car, TSP, FL, and FS. Mean performance and multivariate analysis of 24 cotton genotypes based on various agro-physiological and biochemical parameters suggested that the genotypes FBS-Falcon, Barani-333, JSQ-White Hold, Ghauri, along with crosses FBS-FALCON × JSQ-White Hold, FBG-222 × FBG-333, FBG-222 × Barani-222, and Barani-333 × FBG-333 achieved the maximum values for K^+^/Na^+^, K^+^, TSP, POD, Chlb, CAT, Car, LP, FS, FL, PH, NBP, BW, and SCY under salt stress and declared as salt resistant genotypes. The above-mentioned genotypes also showed relatively higher expression levels of *Ghi-ERF-2D.6* and *Ghi-ERF-7A.6* at 15 dS/m and proved the role of these ERF genes in salt tolerance in cotton. These findings suggest that these genotypes have the potential for the development of salt-tolerant cotton varieties with desirable fiber quality traits.

## Introduction

Soil salinization is a significant obstacle to the sustainable growth of global agriculture and a major threat to the ecological health of the soil. Approximately 37% of all cultivated lands globally are categorized as sodic. It is estimated that almost 50% of the world’s (2.5 billion hectares) of irrigated lands are significantly impacted by salinization^1^. Additionally, every year nearly four million acres of cropland are abandoned due to severe salt damage^2^. Besides the existing salt-affected soil, ongoing secondary salinization in arable land is caused by several factors such as climate change, excessive irrigation, irrigation with saline water, and excessive use of mineral fertilizers^3^. When crop plants experience salt stress, several physiological and biochemical changes occur, including water imbalance, ionic toxicity, nutrient deficiency, oxidative stress and hormonal imbalance which ultimately reduce the crop yield^4^.

Cotton (Gossypium spp.), a main agro-industrial crop, is grown worldwide for its significant production of natural fibers and seed oil. India, China, USA, Brazil, Pakistan, and Australia are among the major cotton-growing countries. Despite being in the top 5 globally, Pakistan has a low yield compared to other leading countries due to various biotic and abiotic factors (drought, heat, and salinity)^5^. Among all these abiotic stresses salinity is a major threat to cotton production. In Pakistan due to competition between cereals and other cash crops, the cultivation of cotton is being moved to saline and alkaline soils. Besides these factors, the increasing demand of natural fiber for textiles, due to population growth and societal reasons, it’s essential to produce high-quality cotton fibers in abundance^6^.

Cotton is tolerant to moderate salinity (7.7 ds/m), but increased soil salinity negatively affects its growth and yield. At the plant level, salinity disrupts various physiological and biochemical processes like ionic toxicity, photosynthesis process, and oxidative injury. As a consequence of this phenomenon, there is inhibition of cell division and expansion in leaf surface area, leading to developmental changes, metabolic adaptations, and decreased stem growth and root cell proliferation. These factors directly contribute to a reduction in the NBP, BW, SCY and fiber quality parameters^7,8^. Salt stress triggers the production of higher levels of reactive oxygen species (ROS) such as superoxide radicals (O_2_^−^), H_2_O_2_, singlet oxygen (^1^O_2_) and hydroxyl radicals (OH^−^), which induce oxidative stress in the plant^9^. The production of ROS at higher level leads to oxidation and damage of plant cell organelles like mitochondria, peroxisomes and chloroplasts. In response, the cell produces antioxidant enzymes such as superoxide dismutase (SOD), peroxidases (POD), catalase (CAT), and non-enzymatic antioxdants^10^ to counter the detrimental effects of oxidation, safeguard the organelles, and uphold the normal functioning of the cell^11^.

Genetic switches also referred to as transcription factors (TFs), attach themselves to specific cis-acting elements, regulating downstream expression of the gene and aiding in the management of stress-induced impairment^12^. In various plant species, many TFs are either induced or suppressed after exposure to salinity stress, facilitating adaptation to saline environments^13^. The *AP2/ERF* gene family is a plant-specific group of TFs characterized by an AP2/ERF-type DNA-binding domain that consists of roughly 60–70 amino acids^14^. This family was initially discovered in the Arabidopsis homeotic gene *APETALA2* (AP2). The *AP2/ERF* gene family is subdivided into four subfamilies, namely *AP2, ERF, RAV*, and *DREB*. The *AP2* subfamily differs from the other three subfamilies in that it possesses double *AP2/ERF* domains, while the other three subfamilies have a single *AP2/ERF* domain^15^. In the past two decades, the *ERF* family of genes has garnered significant importance, as overexpression of *ERF* genes in various plants has been shown to enhance tolerance against abiotic stresses and pathogen resistance in genetically modified plants. In the previous study, based on transcriptome and qRT-PCR analysis, several potential candidate genes (*Ghi-ERF-6D.1, Ghi-ERF-2D.6, Ghi-ERF-12D.13, Ghi-ERF-11D.5*, and *Ghi-ERF-7A.6*) that promote salinity tolerance in upland cotton^13^.

Salinity poses a significant economic and environmental challenge to the global cotton industry, which highlights the need to enhance the salt tolerance of cotton and develop germplasm capable of producing higher yields under changing climatic situations^16^. To mitigate the impact of salinity stress, several strategies have been employed to enhance resilience in cotton plants. Among these, the development of salt-tolerant germplasm is regarded by cotton breeders as the most effective and sustainable solution^16^. Utilizing conventional breeding techniques to screen available germplasm for morphological, physiological, and biochemical traits can leverage natural variations and significantly enhance cotton plant tolerance to salt stress. Measuring the levels of antioxidant enzymes produced in response to salt stress can serve as an effective screening strategy for identifying germplasm with the potential to yield higher crops under saline conditions^8^. Plant’s ability to resist toxic effects of NaCl salinity depends on genetic make-up of plants or variations in physiological processes which enable the plants to cope with salt stress, which include degree of ion exclusion, tolerance to osmotic stress, and tissue tolerance. Moreover, the mechanism of salt tolerance varies with the type of species, type of cultivar of the same species, and plant developmental stage which makes it more complex^17^. Consequently, devising screening techniques and parameters for evaluating salt tolerance becomes a challenging endeavor^18^. Experts emphasize the need to transfer identified physiological and genetic components of salt tolerance into crop cultivars adapted to local environments. Identified salt-tolerant crop cultivars with physiological traits contributing in salt tolerance can be used as donors in breeding for salt tolerance. However, considerable genetic variability is a prerequisite for any specific physiological trait, based on which selection or screening can be made. In this study, we used pure lines which are acclimatized to various ecological zones of Pakistan. The present research aims to achieve three objectives: (i) identification of salt-tolerant cotton cultivars with novel source of salt tolerance from local germplasm, (ii) development of a selection criterion that incorporates agro-physiological and biochemical characters for the production of salt-resistant cotton cultivars, and (iii) evaluation of the expression levels of *Ghi-ERF-2D.6* and *Ghi-ERF-7A.6* genes in salt-tolerant cotton genotypes.

## Materials and methods

The experimental work was carried out on the research premises of FB Genetics, Four Brothers Group, Pakistan during the cropping year 2021. The experimental site is located at 74˚ east longitude and 31˚ north latitude. Under normal field conditions, the F_0_ seeds of eight genotypes were sown and then, in a Line × Tester manner, were crossed. Four genotypes (namely FBS-FALCON, FBS-SMART, FBG-222 and Barani-333) were used as lines and four genotypes (namely JSQ-White Hold, FBG-333, Barani-222 and Ghauri-3) were used as testers (Table 1). When the cotton genotypes were flowering, they were crossed in a line × tester way (4 × 4). To create selfed seeds, certain buds were also wrapped in glassine bags. The mature selfed and crossed bolls were harvested and preserved. Subsequently, the cotton fibers from each group were processed separately using a single roller ginning machine (Testex, Model: TB510C) after being picked. In the next growing season, 24 genotypes (including 8 parents and their 16F1 hybrids) were grown in containers with normal soil (1.9 dS/m) under RCBD with three replications. After the emergence of seedlings, healthy specimens were selected for each genotype in every replication, all of which were subsequently subjected to salt stress through irrigation water with a known EC. The Analysis of soil properties of the experimental site was also conducted (Table 2). The salt stress was induced following a well-established procedure^8^. This method involves gradually increasing the salinity to a target level of ~ 15 dS/m in two distinct phases to avert seedling injury and ensure survival. Sodium chloride (NaCl) was calculated and added to the groundwater using the U.S. Salinity Lab formula^19^.

$${\text{Amount}}\;{\text{of}}\;{\text{sodium}}\;{\text{chloride}}\left( {{\text{g}}/{\text{kg}}} \right) = \frac{Equiv.\;Wt\;of\;NaCl \times Saturation\;Percentage }{{100 \times 1000}} \times TSS$$

**Table 1:** Material used in breeding study.

Code	Genotype name
1	FBS-FALCON
2	FBS-SMART
3	FBG-222
4	Barani-333
5	JSQ-White Hold
6	FBG-333
7	Barani-222
8	Ghauri-3
9	FBS-FALCON × JSQ-White hold
10	FBS-FALCON × FBG-333
11	FBS-FALCON × Barani-222
12	FBS-FALCON × Ghauri-3
13	FBS-SMART × JSQ-White Hold
14	FBS-SMART × FBG-333
15	FBS-SMART × Barani-222
16	FBS-SMART × Ghauri-3
17	FBG-222 × JSQ-White hold
18	FBG-222 × FBG-333
19	FBG-222 × Barani-222
20	FBG-222 × Ghauri-3
21	Barani-333 × JSQ-White hold
22	Barani-333 × FBG-333
23	Barani-333 × Barani-222
24	Barani-333 × Ghauri-3

**Table 2:** Analysis of soil properties used in this study.

Parameters	Unit	Normal field
Textural class (USDA)		Sandy loam
ECe	dS/m	1.9
pHs		8.4
Organic matter	%	0.5
Na^+^	meq/L	3.05

In this equation, TSS (Total Soluble Salts) represents the difference in EC (desired EC—initial EC) multiplied by 12.66, and the saturation percentage of the soil was established at 42%.

During the initial phase, the 2-week-old seedlings were subjected to a salinity level of 7.5 dS/m, which necessitated the addition of approximately 17.74 g of NaCl to each 10 kg pot. Subsequently, in the second phase, the 4-week-old seedlings were subjected to an escalated salinity level of 15 dS/m, which required a further addition of about 23.72 g of NaCl per 10 kg pot. Thus, approximately 41.46 g (17.74 g + 23.72 g) of NaCl was added per 10 kg pot in the two phases to attain the desired salinity level. After reaching this level, no further salt was added. Following this precise modulation of salinity, standard agricultural procedures were then followed for irrigation. This careful approach enabled us to achieve gradual increments in salinity while preventing seedling injury and ensuring survival throughout the experiment.

All agronomic procedures were appropriately carried out from the time of sowing until harvesting. Figure S1 displays the environmental conditions, including temperature, humidity, and rainfall, during crop growth. Upon reaching maturity (i.e., after 140 days of growth and > 80% completion of boll formation), the following parameters were measured from five chosen plants from each replication of both normal and saline treatment: plant height, bolls/plant, boll weight, seed cotton yield/plant, lint%, H_2_O_2_, SOD, POD, CAT, TSP, chlorophyll contents (a & b), carotenoids, fiber strength, fiber length, fiber fineness, Na^+^, K^+^ and K^+^/Na^+^ ratio.

### Ion analysis

The analysis of sodium and potassium ions involved grinding hot air-dried leaves using a pestle and mortar. Concentrated nitric acid and sulfuric acid in a 2:1 ratio were used for the digestion of ground leaves on the hot plate. Then digested samples were cooled down by adding distilled water at room temperature. A flame photometer (410 Flame Photometer) was used to take out readings and then the K^+^ /Na^+^ ratio was calculated.

### Hydrogen peroxide (µmol/g-FW)

Bernt and Bergmeyer^20^ method was used to measure the hydrogen peroxide of treated and control samples. 0.5 g of leaf sample was homogenized using liquid nitrogen for each control and treated group. 1.5 ml of 100 mM potassium phosphate buffer (pH 6.8) was used for the suspension of ground leaves then the suspension was centrifuged at 18,000×*g* for 20 min at 40 °C. The supernatant (0.25 ml) was taken out and mixed with 1.25 ml of peroxidase reagent containing; 40 μg peroxidase/ml, 0.005% (w/v) O-dianizidine and 83 mM potassium phosphate buffer (pH 7.0) at 30 °C to initiate the reaction. After 10 min, the reaction was halted by adding 0.25 ml of 1 N perchloric acid and centrifuging the mixture at 5000×*g* for 5 min. A spectrophotometer (NanoDrop™ 8000 Spectrophotometer Thermo Fisher Scientific, Sweden) was used to measure the absorbance at 436 nm and the amount of H_2_O_2_ was determined based on an extinction coefficient of 39.4 mM^−1^/cm^21^. The SOD activity was measured in terms of enzyme units that inhibited the photochemical reduction of nitro blue tetrazolium (NBT).

### Superoxide dismutase

To quantify SOD, a reaction mixture containing 100 μl of enzyme extract, potassium phosphate buffer (pH 5), 200 μl of methionine, 200 μl of Triton X, 100 μl of NBT, and 800 μl of distilled water was prepared. The mixture was exposed to ultraviolet light for 15 min, followed by the addition of 100 μl of Riboflavin. The absorbance at 560 nm was measured using a spectrophotometer.

### Peroxidase (U/mg protein)

The same enzyme extracted for measuring SOD was used to measure POD values. It is calculated as the number of enzyme units that oxidized guaiacol. The reaction mixture was prepared by mixing 100 μl H_2_O_2_ (40 mM), 100 μl guaiacol (20 mM) with 100 μl of enzyme extract, and 800 μl potassium phosphate buffer (pH 5) in Eppendorf tube and a spectrophotometer was used to measure absorbance at 470 nm wavelength^22^.

### Total Soluble Proteins (mg/g-FW)

To determine the Total Soluble Proteins (TSP), leaf tissue extraction was carried out using vortex and centrifugation in a phosphate buffer (pH 4). Furthermore, a 40 μl portion of the same enzyme extract was mixed with 160 μl of Bradford reagent, added to an ELISA plate, and subjected to spectrophotometer readings at a wavelength of 595 nm absorbance^23^.

### Catalase (U/mg protein)

Catalase activity was determined by measuring the amount of H_2_O_2_ consumed and transformed into H_2_O and O_2_. Spectrophotometer values were recorded at 240 nm absorbance to calculate CAT activity^22^.

### Chlorophyll contents and carotenoids assay

The chlorophyll and carotenoid contents were measured by following^24^. 0.5 g of cotton leaf sample underwent crushing in 8–10 ml of 80% acetone (volume/volume). Subsequently, homogenization occurred through filter paper, and the absorbance of the resulting solution was measured at wavelengths of 645 and 663 nm. The quantification of chlorophyll a, chlorophyll b, and carotenoids followed this procedure.

The chlorophyll a & b and carotenoids were estimated as follows,

$$\begin{gathered} {\text{Chl}}\;{\text{a}}\left( {{\text{mg}}/{\text{g}}\;{\text{FW}}} \right) = \left[ {12.7\left( {{\text{OD}}\;{663}} \right) - 2.69\left( {{\text{OD}}\;{645}} \right)} \right] \times {\text{V/1000}} \times {\text{W}} \hfill \\ {\text{Chl}}\;{\text{b}}\left( {{\text{mg/g}}\;{\text{FW}}} \right) = \left[ {22.9\left( {{\text{OD}}\;{645}} \right) - 4.68\left( {{\text{OD}}\;{663}} \right)} \right] \times {\text{V/1000}} \times {\text{W}} \hfill \\ \end{gathered}$$

$${\text{Carotenoids}}\left( {{\text{mg}}/{\text{g}}\;{\text{FW}}} \right) = {\text{A}}^{{{\text{car}}}} /{\text{Em}} \times {1}00$$

$${\text{A}}^{{{\text{car}}}} = {\text{O}}.{\text{D}}\;{48}0 + 0.{114}\left( {{\text{O}}.{\text{D 663}}} \right){-}0.{638}\left( {{\text{O}}.{\text{D}}\;{645}} \right)$$

where,

W = weight of leaf sample, V = volume of sample, Em = 2500.

### qRT-PCR of Ghi-ERF-2D.6 and Ghi-ERF-7A.6 in tolerant cotton genotypes

The newly emerged leaves of control and stressed (15 dS/m) genotypes at two leaves stage were sampled. The RNA was extracted using a kit protocol (RNAprep Pure Plant Kit by Tiangen, Beijing, China) and the quality and integrity of the RNA were checked using gel electrophoresis and NanoDrop 2000 spectrophotometer (Thermo Scientific, USA) respectively. The cDNA was prepared using (PrimeScript^®^ RT Reagent Kit, Takara Biotechnology Co., China). Three repeats of both genes were used technically. The real-time differential expression of mRNA of both genes was measured using qRT-PCR (Maxima SYBR Green)/ROX qPCR Master mix (2X), cat#K0221, Thermo scientific, USA). Gene normalization was obtained using GAPDH as an internal control. The gene-specific primers were used for the expression of both genes (Table 3). In a PCR reaction gene-specific short-length primers for ERF2 and ERF7 gene were used. A total volume of PCR reaction mixture was prepared in a volume of 25 µl. General PCR conditions using short-length primers were as follows in this study: firstly, one cycle of initial denaturation at 95 °C for 3 min; secondly, 28 cycles of denaturation at 95 °C for 45 s, annealing at 66 °C for 45 s, and extension at 72 °C for 2 min; finally, one cycle of final extension at 72 °C for 10 min.

**Table 3:** The list of the primers.

qReal-time PCR primers		
ERF2-RT-F	AGGTACGGGTTCCTTATGGC	120 bp
ERF2-RT-R	CCGTCTCTGCAGGAACAATC	
ERF7-RT_F	GGGAGTGCATGGGGATTTAC	
ERF7-RT-R	CAATCTTCCGAACTCTTCTGTGT	

### Fiber quality parameters

A seed cotton sample was weighed and subjected to a single roller ginning machine, to separate lint from the seeds. The percentage (%) of the lint was then calculated. This was achieved by dividing the weight of the lint by the weight of the seed cotton in the sample, the obtained value was then finally expressed as a percentage. Furthermore, employing a high-volume instrument (HVI-900, USTER, USA), the resulting lint was given an in-depth analysis to determine characteristics such as fiber strength, fiber fineness and length parameters.

### Statistical analysis

The split-plot analysis of variance (ANOVA) was performed with two factors^25^. The statistical tools prcomp, ggplot2, and Hmisc from R 4.1.1 were used to conduct principal components, and correlation analyses respectively on the mean data. Falconer and Mackay’s method was followed to estimate heritability and genetic advance^26,27^.

### Ethics approval and consent to participate

Study protocol complies with relevant institutional, national, and international guidelines and legislation.

## Results

The results of the ANOVA suggested significant variations among parents and their F_1_ hybrids under salt stress conditions. These differences indicated the existence of genetic diversity in the studied germplasm for salinity tolerance, as shown in (Table 4). Moreover, the treatment and Genotypes × Treatment interaction for all characteristics were highly significant, indicating that all genotypes responded differently to salt stress (Table 4). All cotton genotypes experienced a negative impact on their agronomic and yield-related traits under salt stress. The reduction was observed in agronomic traits such as PH, NBP, BW, SCY, lint%, FL, and FS as shown in (Table 5). Interestingly, fiber fineness (FF) was increased under saline environments. Furthermore, the mean values for H_2_O_2_, Na^+^, CAT, POD, and SOD were increased under salt stress, while the mean values for K^+^/Na^+^, K^+^, TSP, Chla and b were decreased under saline conditions.

**Table 4:** ANOVA split plot design for different agronomic and physiological traits.

SOV	Replication	Treatments	Error A	Genotypes	Treatments × genotypes	Error B
DF	2	1	2	23	23	92
Boll weight	0.14	66.59**	0.05	2.04**	9.94*	0.14
Catalase	23.68	2218.17*	34.99	577.3**	9.37**	10.30
Carotenoids	0.04	4.62**	0.00	0.16**	5.74**	0.02
Chlorophyll a	0.13	17.32**	0.11	0.41**	2.86**	0.07
Chlorophyll b	0.003	1.20**	0.00	0.04**	4.17**	0.00
Fiber fineness	0.06	81.48**	0.23	5.24**	7.18**	0.14
Fiber length	0.09	5335.82**	0.28	151.9**	14.64**	4.89
Fiber strength	2.43	3235.33**	1.63	127.1*	8.93**	5.98
Hydrogen peroxide	0.00	2.84**	0.02	0.2**	1.32	0.02
Potassium	104.10	12,511.4*	23.50	1166.1**	6.29**	42.70
Potassium-to-sodium ratio	1.00	76.07**	0.01	1.17 **	10.94**	0.14
Lint percentage	9.70	11,507.4**	19.10	356.4**	14.31**	14.40
Sodium	120.04	7863.99**	5.16	89.38**	7.23**	20.32
Number of bolls	7.34	4841.84**	3.55	108.73**	8.11**	9.96
Plant height	62.80	11,612.8**	38.20	429.8**	5.32**	30.20
Peroxidase	11.82	762.86**	1.48	165.7**	6.32**	4.11
Seed cotton yield	23.92	5451.73**	40.26	130.77**	14.36**	6.27
Superoxide dismutase	0.83	304.39**	0.87	22.7**	17.96**	0.77
Total soluble proteins	772.40	71,679.4**	53.40	73,718.5**	5.36**	308.20

*Significant at the 0.05 level.

**Significant at the 0.01 level.

**Table 5:** Genetic components of variability, genetic advance percentage means and heritability (broad sense) estimates for studied traits across control and salt stress conditions.

SOV	Treatment	Min	Max	Mean	h^2^b	GAM
Boll weight (g)	Control	2.0973	4.838	3.4328	30.61	31.39
	Stress	0.121	4.129	2.0733	89.86	22.19
Catalase (U/mg protein)	Control	14.05	45.6091	29.5031	84.88	20.77
	Stress	16.4609	59.7862	37.3531	93.98	44.39
Carotenoids (mg/g FW)	Control	0.43	1.49	0.7058	47	39.57
	Stress	0.0863	1.2997	0.3475	83.98	16.43
Chlorophyll a (mg/g FW)	Control	0.36	1.985	1.2565	14.37	40.35
	Stress	0.0607	1.6396	0.5637	75.45	33.07
Chlorophyll b (mg/g FW)	Control	0.2059	0.6768	0.3822	12.90	44.47
	Stress	0.0145	0.533	0.1993	90.11	38.05
Fiber fineness (µg/inch)	Control	0.3462	4.4903	2.1767	93.14	18.13
	Stress	2.05	5.15	3.6812	73.79	20.48
Fiber length (mm)	Control	16.29	31.785	26.3596	78.50	14.58
	Stress	2.93	28.42	14.1857	90.92	11.19
Fiber strength (g/tex)	Control	17.672	29.814	23.7402	25.41	21.09
	Stress	4.148	27.932	14.2603	93.25	12.27
Hydrogen peroxide (µmol/g FW)	Control	0.303	1.166	0.6221	68.85	36.76
	Stress	0.4579	1.3713	0.9033	62.50	38.25
Potassium (mM)	Control	135.34	179.78	156.89	73	13.87
	Stress	100.29	178.67	138.25	88	26.30
Potassium-to-sodium ratio (%)	Control	2.77	7.22	4.12	60	21.41
	Stress	1.68	5.09	2.67	86	30.74
Lint percentage (%)	Control	32.1954	57.5275	44.502	16.01	13.55
	Stress	7.9245	49	26.6242	94.80	11.48
Sodium (mM)	Control	20.4	56.06	38.9845	65.03	21.92
	Stress	33.18	69.98	53.7644	59.49	17.39
Number of bolls	Control	13	32	24.6389	18.82	36.23
	Stress	3	27	13.0417	88.20	22.56
Plant height (cm)	Control	50.29	93.22	66.941	19.64	12.05
	Stress	28.36	70.49	48.9806	90.56	14.89
Peroxidase (U/mg protein)	Control	5.25	19.7774	11.9577	81.13	54.77
	Stress	7.1406	31.7854	16.5612	90.14	82.40
Seed cotton yield (g/plant)	Control	22.32	32.658	27.8429	81.07	29.61
	Stress	5.4634	30.3084	15.5381	73.27	61.97
Superoxide dismutase (U/mg protein)	Control	3.04	7.904	5.5599	55.94	19.11
	Stress	4.1312	16.845	8.4673	90.63	77.37
Total soluble proteins (mg/g FW)	Control	336.8	739.6	553.3943	97.07	37.43
	Stress	288	736.8	508.7726	97.96	48.25

The GAM was classified into three categories: low (0–10%), moderate (10–20%), and high (20% or higher). In terms of heritability, values exceeding 80% were considered very high, values ranging from 60 to 79% were moderately high, values between 40 and 59% were medium, and values lower than 40% were regarded as low^28^. Under saline conditions, high genetic advance as a percentage of the mean (GAM) was observed for BW, CAT, Chla, Chlb, FF, NBP, POD, SCY, SOD and TSP whereas moderate GAM was observed for PH, LP, Na^+^, K^+^, FS, FL, and Car. The heritability estimates for BW, CAT, Car, Chlb, FL, FS, K^+^, K^+^/Na^+^, LP, NBP, PH, POD, SOD, and TSP, were very high whilst moderate heritability estimates were observed for SCY, Na^+^, H_2_O_2_, FF, and Chla under saline conditions (Table 5).

### Mean performance of agronomic, and fiber quality traits

**Figure 1:**
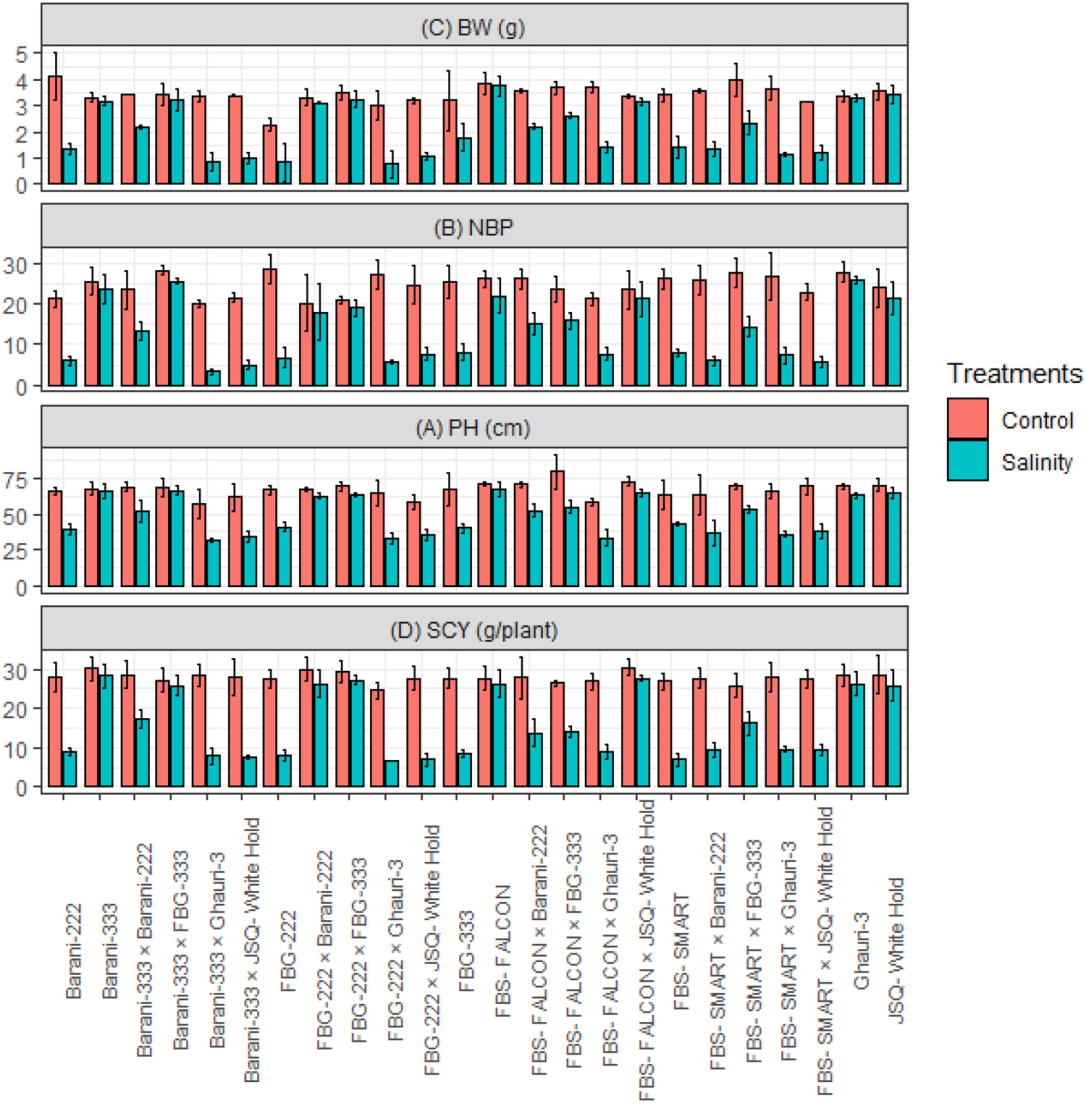
Mean performance of cotton genotypes under control and salinity stress for (**A**) BW, Boll weight (g); (**B**) NBP, Number of bolls; (**C**) PH, Plant height (cm); (**D**) SCY, Seed cotton yield (g/plant).

**Figure 2:**
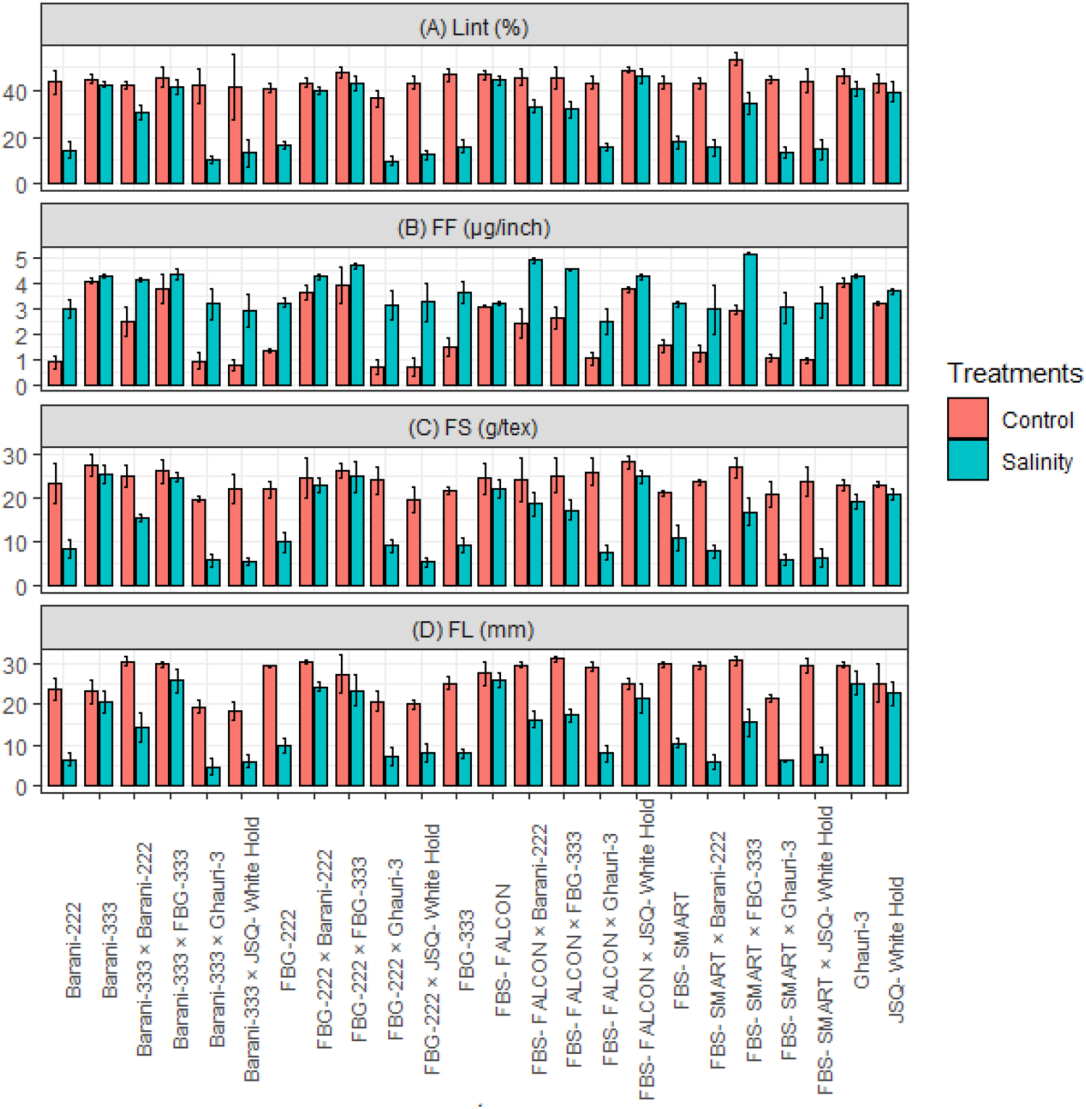
Mean performance of cotton genotypes under control and salinity stress for (**A**) Lint (%) = Lint percentage; (**B**) FF , Fiber fineness (µg/inch); (**C**) FS, Fiber strength (g/tex); (**D**) FL, Fiber length (mm).

Under the influence of salt stress conditions (15 ds/m), the growth and agronomic characteristics of the examined genotypes were negatively impacted. Notably, the effects of salt stress were particularly evident in terms of plant height, the number of bolls per plant, and boll weight, lint percentage, fiber fineness, and fiber strength for all the genotypes studied along with crosses, as depicted in (Figs. 1 and 2). Under salt stress conditions (15 ds/m), certain genotypes and crosses demonstrated remarkable growth and yield performance compared to other studied genotypes. Genotypes FBS-Falcon, Barani-333, JSQ-White Hold, Ghauri, along with crosses FBS-FALCON × JSQ-White Hold, FBG-222 × FBG-333, FBG-222 × Barani-222, and Barani-333 × FBG-333 displayed notable resilience and exhibited superior performance for following traits such as PH, NBP, BW, SCY, lint%, FL, FF, and FS. Conversely, the genotypes (FBS-Smart, FBG-222, FBG-333, and Barani-222), and crosses (FBS-FALCON × Ghauri-3, FBS-SMART × JSQ-White Hold, FBS-SMART × Barani-222, FBS-SMART × Ghauri-3, FBG-222 × JSQ-White Hold, FBG-222 × Ghauri-3, Barani-333 × JSQ-White Hold, and Barani-333 × Ghauri-3) demonstrated lower performance for agronomic and fiber quality traits under salt stress conditions. Notably, among the studied crosses (FBS-FALCON × FBG-333, FBS-FALCON × Barani-222, FBS-SMART × FBG-333, and Barani-333 × Barani-222), revealed moderate performance for PH, NBP, BW, SCY, lint%, FL, FF, and FS under salt stress conditions. These specific crosses demonstrated moderate resilience to the adverse effects of salt stress, indicating their potential for further exploration and breeding programs (Figs. 1 and 2).

### Biochemical traits

**Figure 3:**
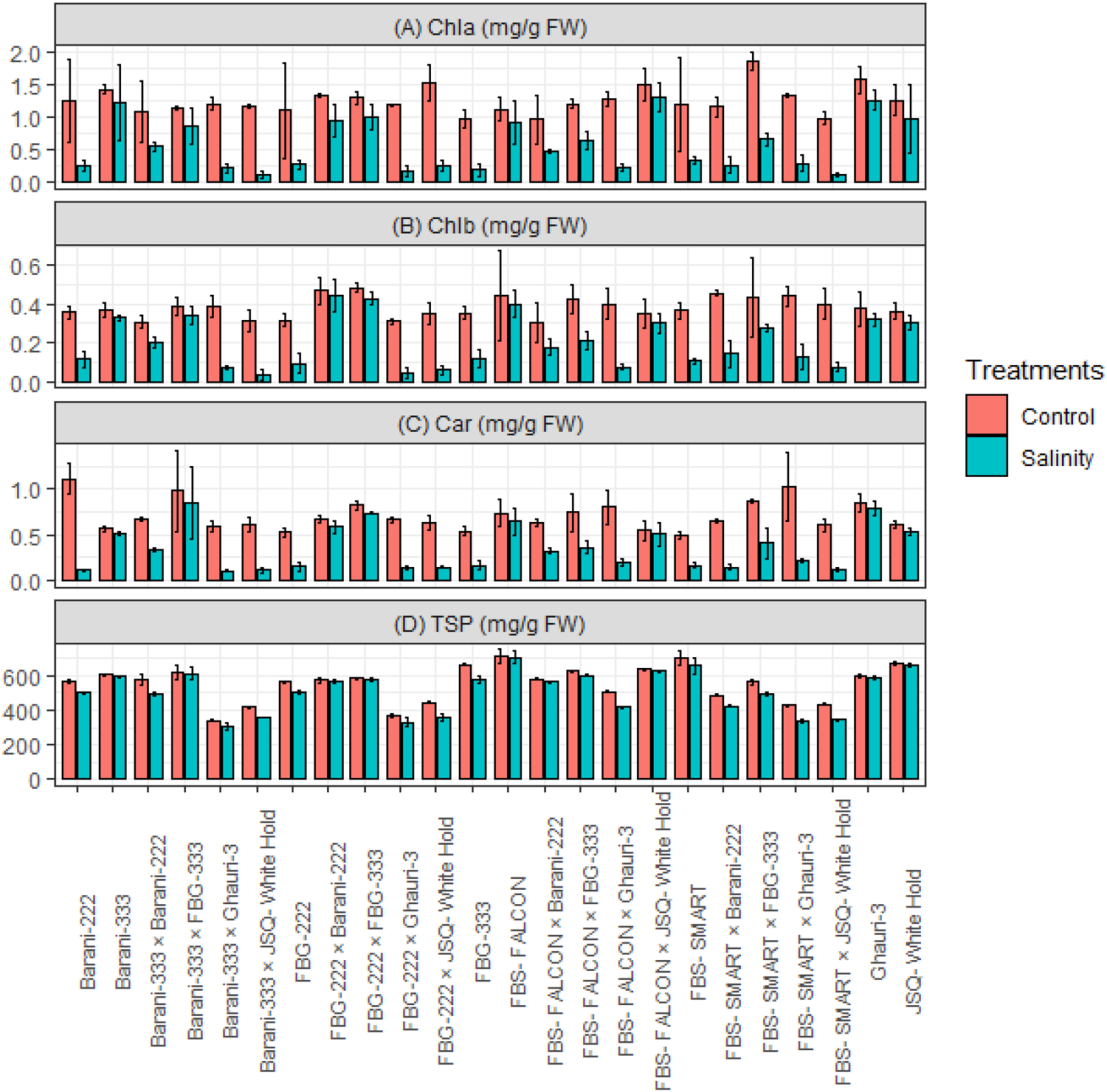
Mean performance of cotton genotypes under control and salinity stress for (**A**) Chla, Chlorophyll a (mg/g FW); (**B**) Chlb, Chlorophyll a (mg/g FW); (**C**) Car, Carotenoids (mg/g FW); (**D**) TSP, Total soluble proteins (mg/g FW).

The biochemical traits such as chlorophyll a (Chla), chlorophyll b (Chlb), total soluble proteins (TSP), and carotenoid (CAR) exhibited a statistically significant decrease in all genotypes under salt stress (15 ds/m) (Fig. 3). The application of salt stress treatment led to a more significant reduction in Chla, Chlb, TSP, and CAR levels in specific genotypes and crosses, such as FBS-Smart, FBG-222, FBG-333, Barani-222, FBS-FALCON × Ghauri-3, FBS-SMART × JSQ-White Hold, FBS-SMART × Barani-222, FBS-SMART × Ghauri-3, FBG-222 × JSQ-White Hold, FBG-222 × Ghauri-3, Barani-333 × JSQ-White Hold, and Barani-333 × Ghauri-3. However, the genotypes FBS-Falcon, Barani-333, JSQ-White Hold, Ghauri, FBS-FALCON × JSQ-White Hold, FBG-222 × FBG-333, FBG-222 × Barani-222, and Barani-333 × FBG-333 exhibited the ability to maintain moderate level of Chla, Chlb, TSP, and CAR (Fig. 3). Furthermore, certain genotypes and crosses, such as FBS-FALCON × FBG-333, FBS-FALCON × Barani-222, FBS-SMART × FBG-333, and Barani-333 × Barani-222 exhibited less reduction in Chla, Chlb, TSP, and CAR level compared to susceptible genotypes (Fig. 3).

**Figure 4:**
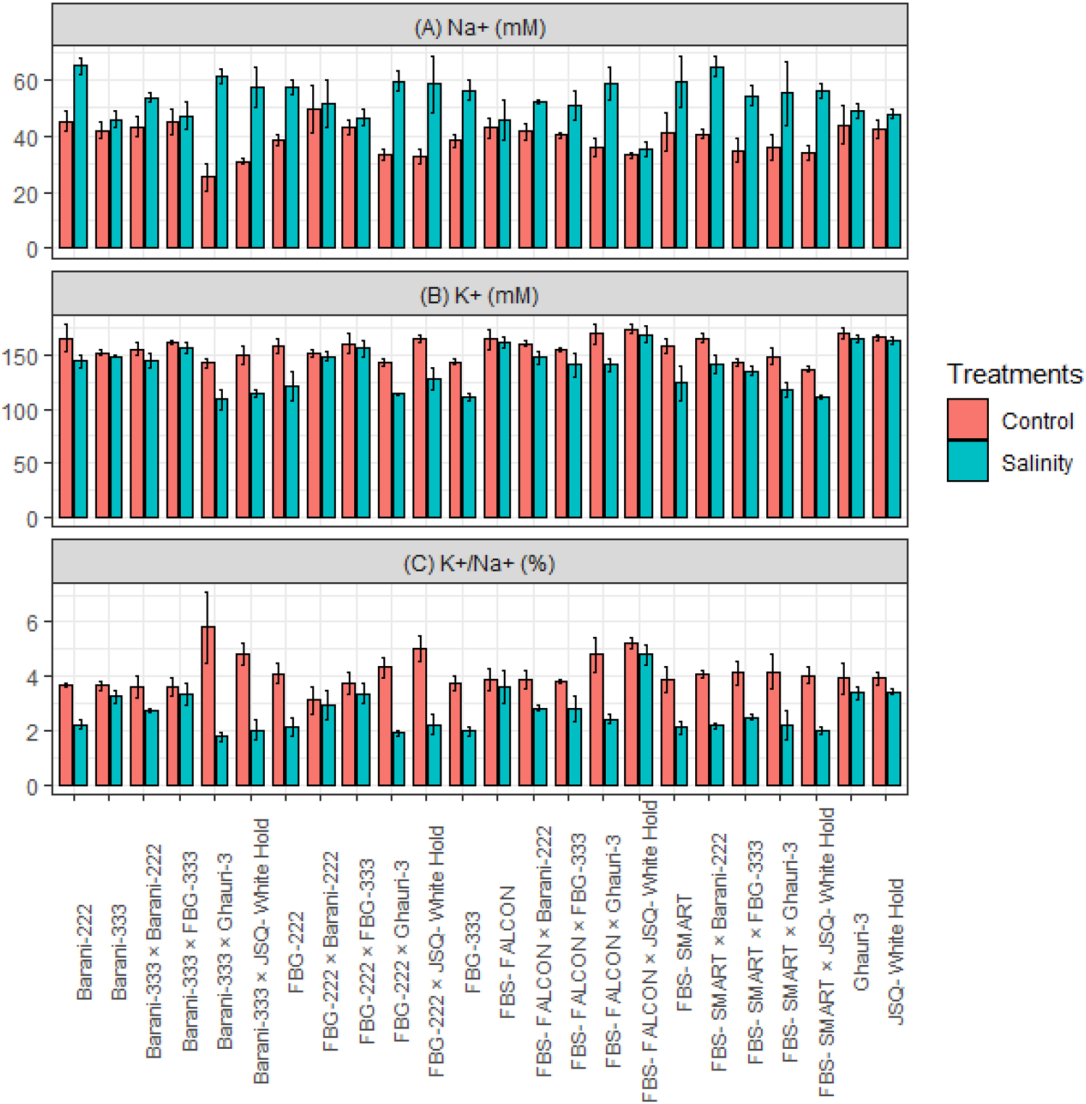
Mean performance of cotton genotypes under control and salinity stress for (**A**) Na^+^, Sodium contents (**B**) K^+^, Potassium contents (**C**) K^+^/Na^+^, Potassium-to-sodium ratio (%).

The application of NaCl treatment had a significant impact on the ionic homeostasis of plants, particularly on Na^+^, K^+^, and the K^+^/Na^+^ ratio. Notably, the Na^+^ content in NaCl-treated plants was found to be significantly higher compared to the control group, indicating an increase in sodium accumulation as a result of the treatment. The mean graph of (Fig. 4) depicted that certain genotypes and crosses, including FBS-Falcon, Barani-333, JSQ-White Hold, Ghauri, FBS-FALCON × JSQ-White Hold, FBG-222 × FBG-333, FBG-222 × Barani-222, and Barani-333 × FBG-333 lower increase in sodium contents compared other genotypes. Compared to other genotypes, these genotypes also revealed less reduction in potassium contents. Consequently, these genotypes and crosses maintained a higher ratio of potassium to sodium content, similar to that of the control group and performed well for biochemical, agronomic and fiber quality characters under saline environments (Fig. 4). However, specific crosses such as FBS-FALCON × FBG-333, FBS-FALCON × Barani-222, FBS-SMART × FBG-333, and Barani-333 × Barani-222 demonstrated a moderate accumulation of Na^+^, and exhibited moderate potassium to sodium ratio. The mean graph reveals that the genotypes and crosses, including FBS-Smart, FBG-222, FBG-333, Barani-222, FBS-FALCON × Ghauri-3, FBS-SMART × JSQ-White Hold, FBS-SMART × Barani-222, FBS-SMART × Ghauri-3, FBG-222 × JSQ-White Hold, FBG-222 × Ghauri-3, Barani-333 × JSQ-White Hold, and Barani-333 × Ghauri-3 exhibited higher levels of Na^+^, along with a lower potassium to sodium ratio under salt stress conditions and declared as salt susceptible genotypes (Fig. 4).

**Figure 5:**
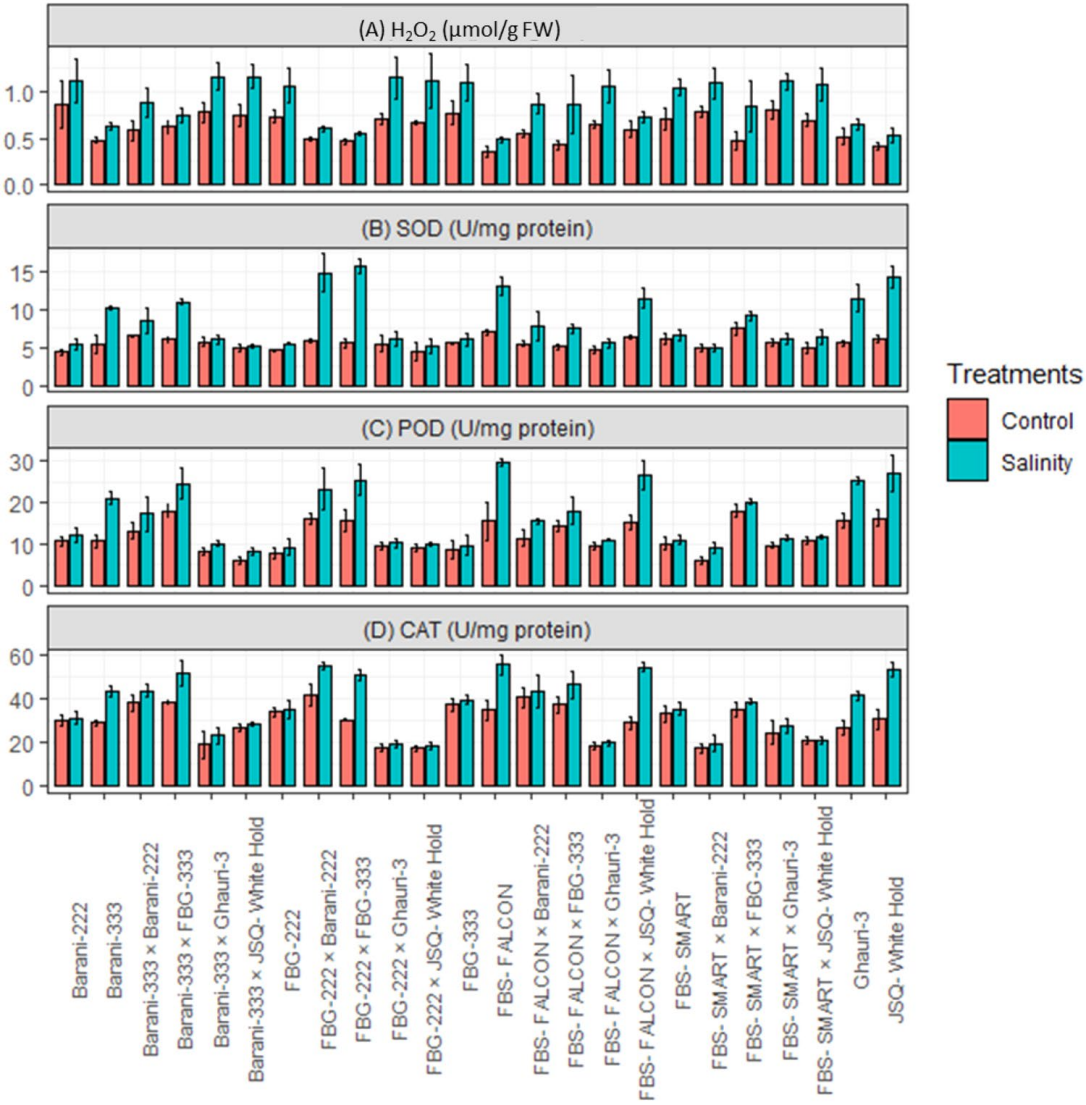
Mean performance of cotton genotypes under control and salinity stress for (**A**) H_2_O_2_, Hydrogen peroxide (µmol/g FW) (**B**) SOD, Superoxide dismutase (U/mg protein) (**C**) POD, Peroxidase (U/mg protein) (**D**) CAT, Catalase (U/mg protein).

Under salt treatment (15 ds/m), the cotton genotypes displayed a significant rise in the levels of reactive oxygen species (ROS). In contrast to the control group, all genotypes exhibited noticeable increases in the levels of H_2_O_2_, indicating the presence of oxidative stress induced by salt stress. However, it is noteworthy that susceptible genotypes and specific crosses, including FBS-Smart, FBG-222, FBG-333, Barani-222, FBS-FALCON × Ghauri-3, FBS-SMART × JSQ-White Hold, FBS-SMART × Barani-222, FBS-SMART × Ghauri-3, FBG-222 × JSQ-White Hold, FBG-222 × Ghauri-3, Barani-333 × JSQ-White Hold, and Barani-333 × Ghauri-3, exhibited greater levels of oxidative damage and showed higher value for H_2_O_2_ under saline conditions (Fig. 5A). This observation suggests that these particular cultivars possess lower tolerance to salt stress when compared to tolerant genotypes such as FBS-Falcon, Barani-333, JSQ-White Hold, Ghauri, FBS-FALCON × JSQ-White Hold, FBG-222 × FBG-333, FBG-222 × Barani-222, and Barani-333 × FBG-333. In contrast, moderate levels of H_2_O_2_ were observed in the following crosses such as FBS-FALCON × FBG-333, FBS-FALCON × Barani-222, FBS-SMART × FBG-333, and Barani-333 × Barani-222 (Fig. 5A). These genotypes also showed a moderate level of SOD, POD, and CAT activities under saline environmental conditions. Our findings indicate that under the salt stress condition of (15 ds/m), there was a higher level of SOD, CAT, and POD activity observed compared to the control group. The tolerant genotypes exhibited optimal levels of SOD, CAT, and POD activity under saline conditions. The tolerant genotypes FBS-Falcon, Barani-333, JSQ-White Hold, Ghauri, FBS-FALCON × JSQ-White Hold, FBG-222 × FBG-333, FBG-222 × Barani-222, and Barani-333 × FBG-333 revealed high performance for SOD, POD and CAT as compared to moderately tolerant and susceptible genotypes. The susceptible genotypes FBS-Smart, FBG-222, FBG-333, Barani-222, FBS-FALCON × Ghauri-3, FBS-SMART × JSQ-White Hold, FBS-SMART × Barani-222, FBS-SMART × Ghauri-3, FBG-222 × JSQ-White Hold, FBG-222 × Ghauri-3, Barani-333 × JSQ-White Hold, and Barani-333 × Ghauri-3 showed lesser increase in SOD, POD and CAT activities under saline conditions (Fig. 5B, C, D).

### Correlation analysis

**Figure 6:**
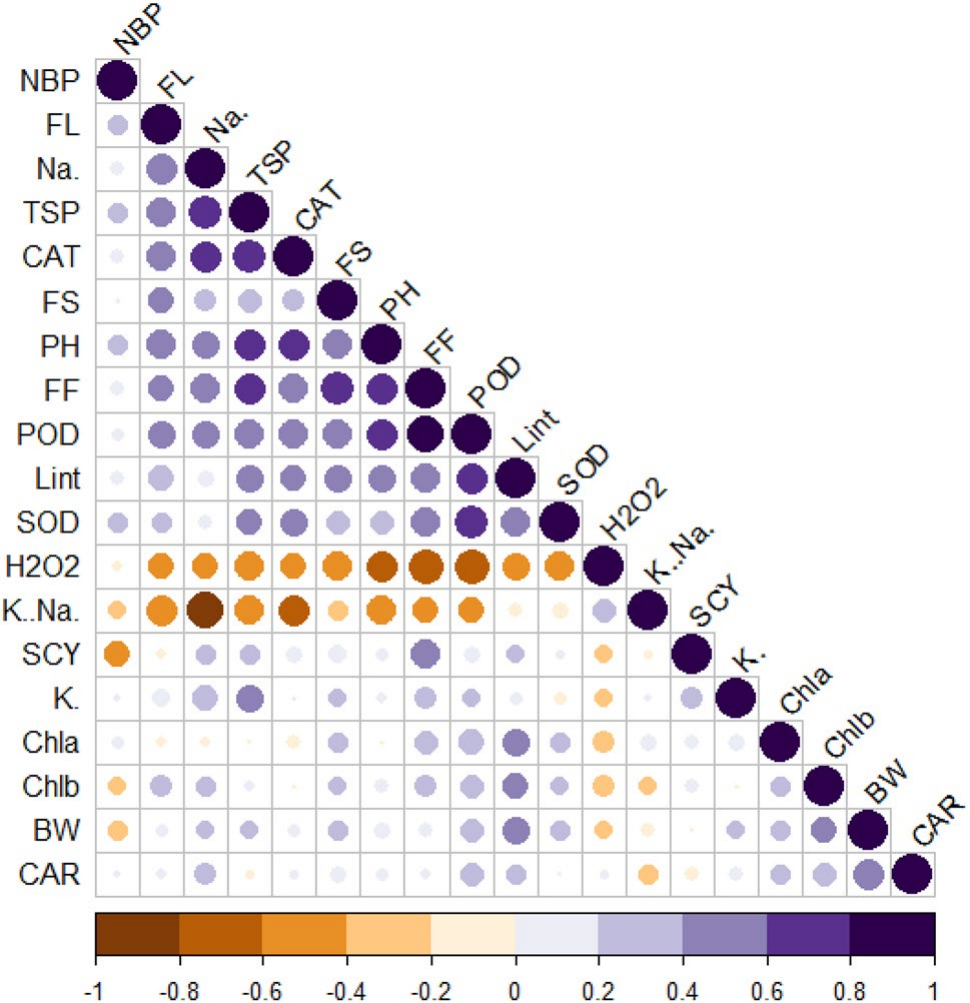
Corplot for various traits under control conditions. BW, Boll weight; NBP, Number of bolls; PH, Plant height; SCY, Seed cotton yield; Lint (%), Lint percentage; FF, Fiber fineness; FS, Fiber strength; FL, Fiber length; Chla, Chlorophyll a; Chlb, Chlorophyll a (mg/g FW); (**C**) Car, Carotenoids (mg/g FW); (**D**) TSP, Total soluble proteins; Na^+^, Sodium; K^+^, Potassium; K^+^/Na^+^, Sodium-to-potassium ratio; H_2_O_2_, Hydrogen peroxide; SOD, Superoxide dismutase; POD, Peroxidase; CAT, Catalase.

**Figure 7:**
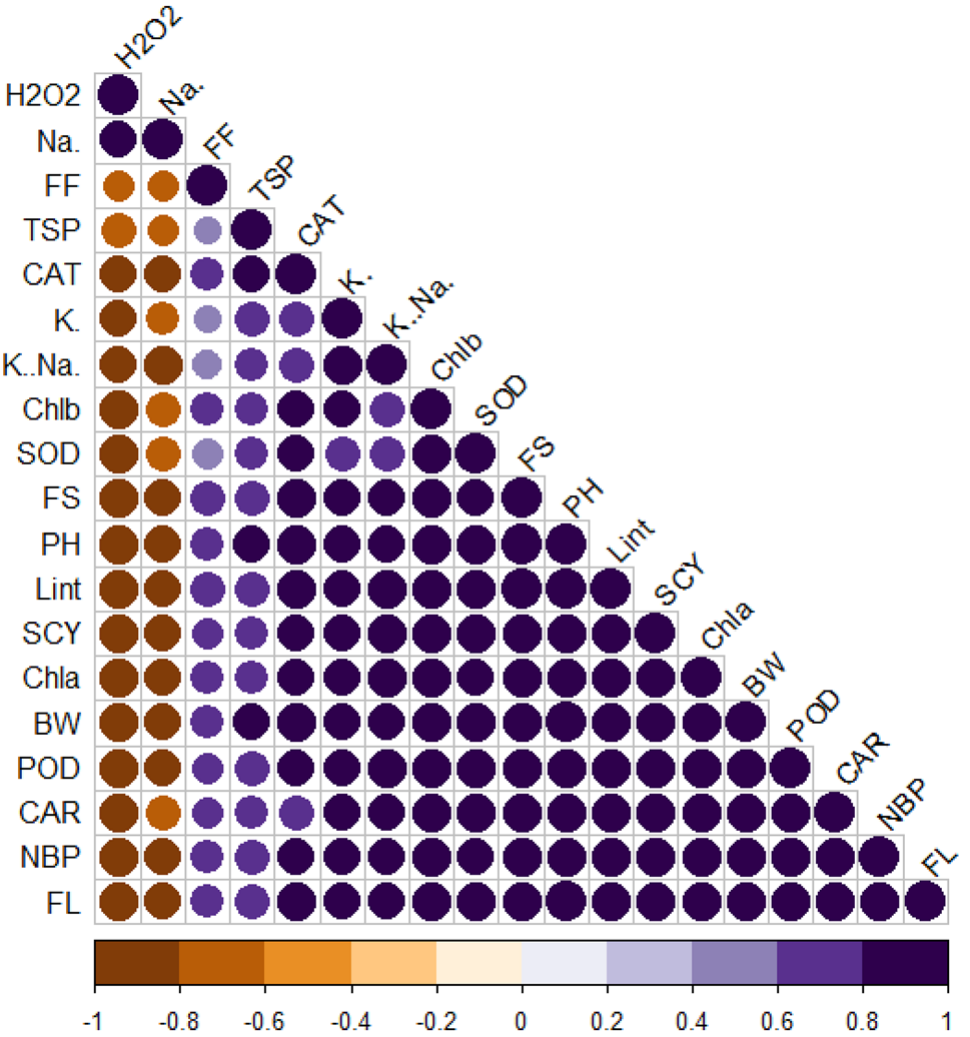
Corplot for various traits under salinity stress. BW, Boll weight; NBP, Number of bolls; PH, Plant height; SCY, Seed cotton yield; Lint (%), Lint percentage; FF, Fiber fineness; FS, Fiber strength; FL, Fiber length; Chla, Chlorophyll a; Chlb, Chlorophyll a (mg/g FW); (**C**) Car, Carotenoids (mg/g FW); (**D**) TSP, Total soluble proteins; Na^+^, Sodium; K^+^ , Potassium; K^+^/Na^+^, Sodium-to-potassium ratio; H_2_O_2_, Hydrogen peroxide; SOD, Superoxide dismutase; POD, Peroxidase; CAT, Catalase.

The relationship between various morpho-physiological and biochemical traits is crucial in determining the most suitable genotype under both normal and saline environments. The Pearson correlation coefficients were computed separately for normal and saline conditions. Under normal conditions, FL, Na^+^, TSP, CAT, FS, PH, FF, POD, and SOD showed positive association with each other. Lint % revealed a significant positive relationship with FL, TSP, CAT, FS, FF, POD, Chla, Chlb, BW, and CAR contents under control conditions (Fig. 6). Under salt stress conditions, H_2_O_2_ and Na^+^ revealed a significant negative relationship with all morphological, agronomical (SCY, BW, NBP, and LP), fiber quality traits (FL, FS, & FF) and physiological characters (CAT, TSP, SOD, POD, Car, chlorophyll contents, K^+^/Na^+^, and K^+^ respectively (Fig. 2). Under salt stress environments all agronomic characters (SCY, BW, NBP, and LP) revealed positive relationship with physiological traits CAT, TSP, POD, Car, chlorophyll contents, K^+^/Na^+^, and K^+^ respectively. The fiber quality parameters showed a positive association with all antioxidant and biochemical traits under salt stress conditions (Fig. 7).

### Principal component analysis (PCA) for agro-physiological, biochemical, and fiber quality traits

**Figure 8:**
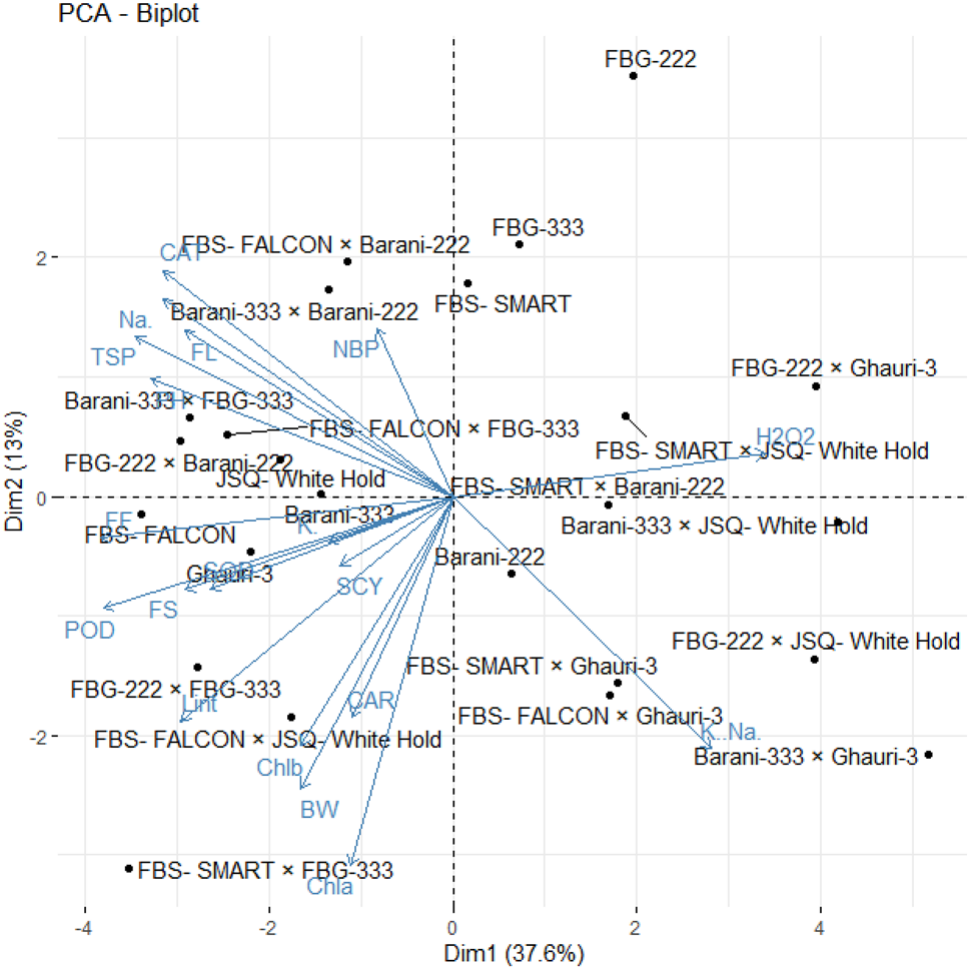
Principal component analysis of cotton genotypes for various traits under control condition. BW, Boll weight; NBP, Number of bolls; PH, Plant height; SCY, Seed cotton yield; Lint (%), Lint percentage; FF, Fiber fineness; FS, Fiber strength; FL, Fiber length; Chla, Chlorophyll a; Chlb, Chlorophyll a (mg/g FW); (**C**) Car, Carotenoids (mg/g FW); (**D**) TSP, Total soluble proteins; Na^+^, Sodium; K^+^, Potassium; K^+^/Na^+^ , Sodium-to-potassium ratio; H_2_O_2_, Hydrogen peroxide; SOD, Superoxide dismutase; POD, Peroxidase; CAT, Catalase.

PCA is a statistical approach for analyzing and simplifying complex datasets. We used PCA to evaluate the correlation among variables and the genetic diversity in studied accessions and their association with studied characters under normal and saline conditions. The PC-1 and PC-2 explained 37.6% and 13% of the whole variation under control conditions, respectively (Fig. 8). The relative distance of variables from the origin in PC1 and PC2 showed how much each variable contributes to the total variation among the studied accessions. Under normal conditions, the biplot demonstrated that genotypes of quadrant-IV had high potential for SCY, LP, BW, chlorophyll contents, POD, FS, SOD, K^+^, and carotenoid contents. The genotypes of quadrant-III performed better for NBP, TSP, CAT, PH, Na^+^, and FL (Fig. 8). The genotypes of quadrant-I revealed good performance for potassium to sodium ratio whereas the genotypes of quadrant-II showed optimal performance for H_2_O_2_ under control conditions (Fig. 8).

**Figure 9:**
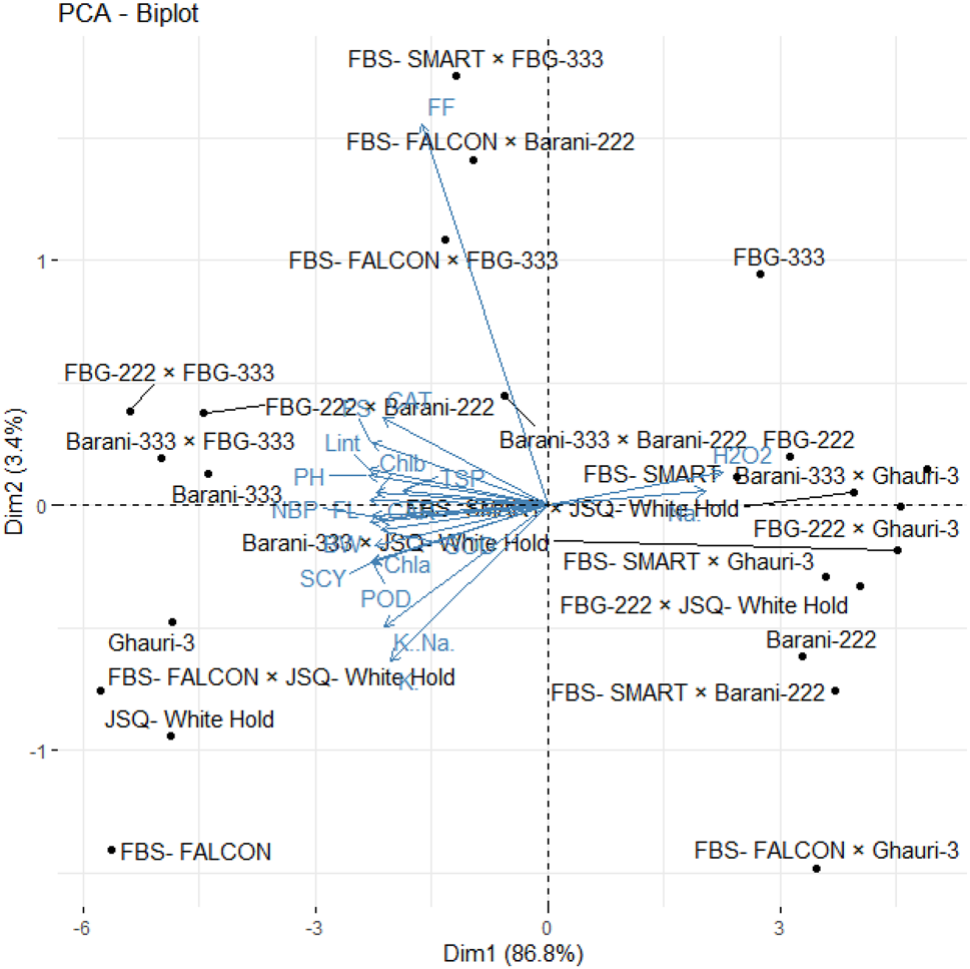
Principal component analysis of cotton genotypes for various traits under salinity stress. BW, Boll weight; NBP, Number of bolls; PH, Plant height; SCY, Seed cotton yield; Lint (%), Lint percentage; FF, Fiber fineness; FS, Fiber strength; FL, Fiber length; Chla, Chlorophyll a; Chlb, Chlorophyll a (mg/g FW); (**C**) Car, Carotenoids (mg/g FW); (**D**) TSP, Total soluble proteins; Na^+^ , Sodium; K^+^, Potassium; K^+^/Na^+^, Sodium-to-potassium ratio; H_2_O_2_, Hydrogen peroxide; SOD, Superoxide dismutase; POD, Peroxidase; CAT, Catalase.

Under saline conditions, the biplot of PC-1 and PC-2, together account for 90.20% of the total variation. In the biplot, traits close to each other were positively associated with each other (Fig. 9). Under salt stress conditions, the biplot of PC1 and PC2, revealed a positive association between Na^+^ and H_2_O_2_ and the genotypes FBG-222, FBS-SMART, FBG-333, Barani-333 × JSQ-White Hold, FBS-SMART × Ghauri-3, Barani-333 × Ghauri-3, FBG-222 × JSQ-White Hold, FBS-SMART × JSQ-White Hold, FBS-SMART × Ghauri-3, FBS-FALCON × Ghauri-3, Barani-222, and FBS-SMART × Barani-222 showed a positive relationship with these traits and declared as salt susceptible. Whereas the genotypes FBS-FALCON × JSQ-White Hold, FBS-FALCON, JSQ-White Hold, FBG-222 × FBG-333, FBG-222 × Barani-222, Barani-333, Ghauri-3, and Barani-333 × FBG-333 revealed a positive correlation with K^+^/Na^+^, K^+^, POD, SOD, CAT, TSP, Chla, Chlb, CAR, LP, FS, FL, FS, NBP, BW, and SCY and declared as salt tolerant (Fig. 9). The genotypes FBS-FALCON × FBG-333, FBS-FALCON × Barani-222, and FBS-SMART × FBG-333 showed significant association with FF under saline conditions. The insights obtained from PCA can be utilized to obtain valuable outcomes (Fig. 9).

### Examination of the expression of ERF genes (*Ghi-ERF-2D.6* and *Ghi-ERF-7A.6*) by qPCR

**Figure 10:**
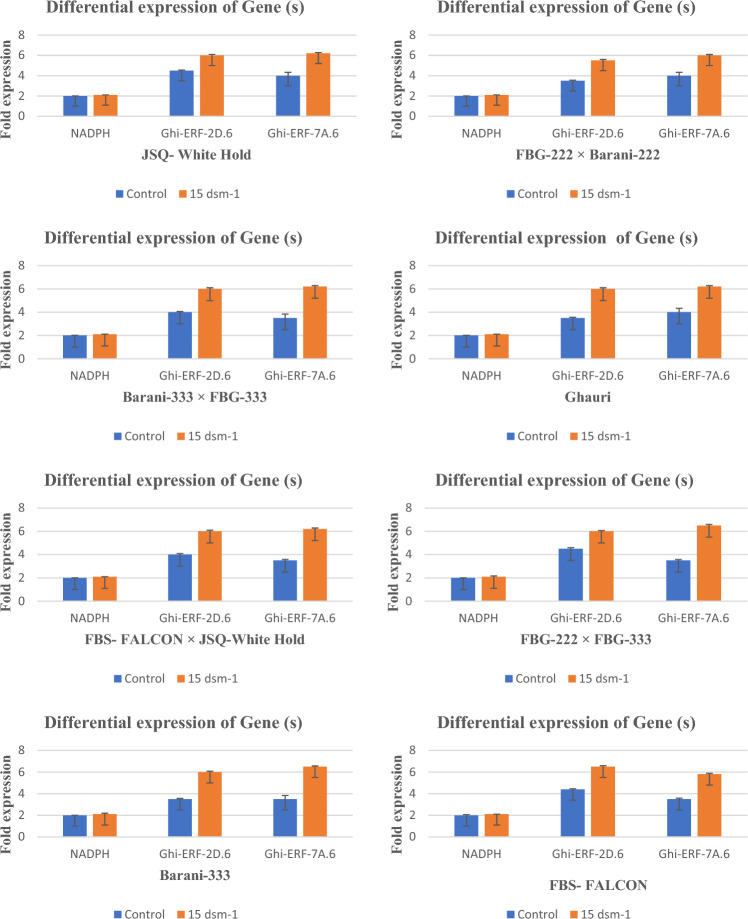
Relative expression patterns of Ghi-ERF-2D.6 and Ghi-ERF-7A.6 genes under normal and salinity stress (15 dS/m NaCl) treatment analyzed by qRT-PCR.

**Figure 11:**
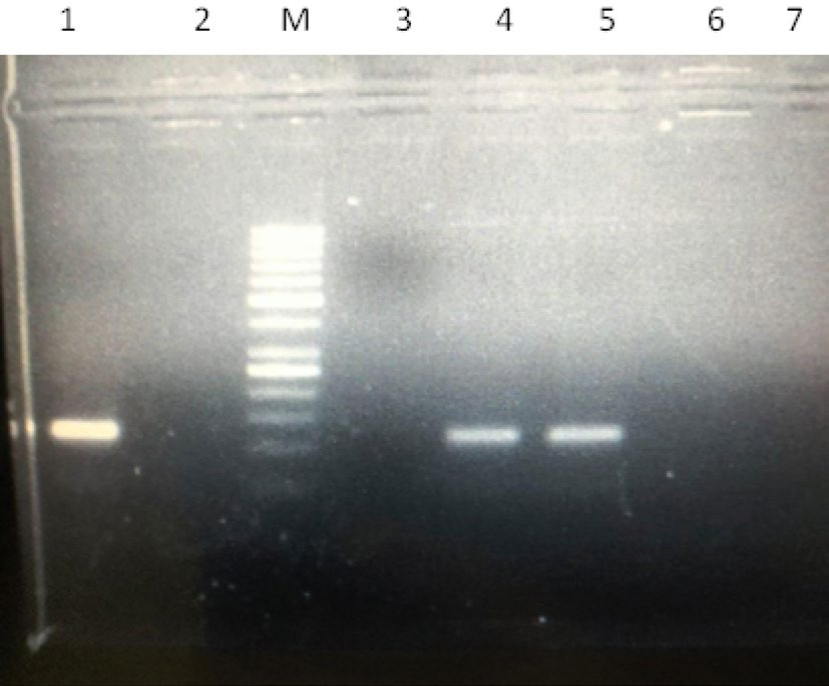
Detection of genes (*ERF2* and *ERF7*) using short length primers; Lane M: shows molecular weight marker; Lane 1: shows positive control; lane 2, 3, 6, 7: shows negative control; lane 4: shows *ERF2*; Lane 5: shows *ERF7* corresponding a size of 120 bp.

The quantitative measurement of mRNA expression in the leaf of genotypes at salt stress levels of control and 15 ds/m was performed. As an internal control of gene normalization, the *GAPDH* was employed. In Fig. 10, the genes *Ghi-ERF-2D.6* and *Ghi-ERF-7A.6* showed relatively higher expression at 15 ds/m. In genotype JSQ-white Hold, the expression of *Ghi-ERF-2D.6* is 1.5 folds higher at 15  ds/m than control whereas the expression of *Ghi-ERF-7A.6* is 2.2 folds higher at 15 ds/m than control. The genotype Barani-333 × FBG-333 showed 2 folds higher expression of *Ghi-ERF-2D.6* at 15 ds/m than control whereas the expression of *Ghi-ERF-7A.6* is 2.7 folds higher at 15 ds/m than control. The expression of both *Ghi-ERF-2D.6* and *Ghi-ERF-7A.6* in genotype FBG-222 × Barani-222 was measured 2 folds higher at 15 ds/m than control. In genotype Ghauri, the expression of *Ghi-ERF-2D.6* and *Ghi-ERF-7A.6* was 2.5 folds and 2.2 folds higher at 15 ds/m than control respectively. The genotype FBG-222 × FBG-333 showed 1.5 folds and 3 folds higher expression of *Ghi-ERF-2D.6* and *Ghi-ERF-7A.6* at 15 ds/m than control respectively (Fig. 10). In genotype FBS-FALCON × JSQ-white Gold, the expression of *Ghi-ERF-2D.6* is 2 folds higher whereas the expression of *Ghi-ERF-7A.6* is 2.7 folds higher at 15 ds/m than control respectively. The genotype Barani-333 showed 2.5 folds and 3 folds higher expression of *Ghi-ERF-2D.6* and *Ghi-ERF-7A.6* at 15 ds/m than control respectively. The expression of *Ghi-ERF-2D.6* and *Ghi-ERF-7A.6* in genotype FBS-FALCON was measured 2.1 folds and 2.3 folds higher at 15 ds/m than control respectively. The expression level of both the genes was increased at 15 ds/m and showed significant tolerance against salt stress (Fig. 10). The PCR reaction using short length primers was run to detect the correct size of the gene, which corresponds 120 bp (Fig. 11).

## Discussion

Cotton is an economically important crop that is grown worldwide for its fiber and seed oil. However, cotton growth and productivity are often limited by various abiotic stresses, including salt stress^4^. Salt stress can have a significant impact on cotton physiology, leading to reduced growth, disrupted water and ionic balance, oxidative stress, and decrease in yield. Understanding these physiological effects is critical for developing strategies to mitigate the negative effects of salt stress and improve cotton productivity in salt-affected soils^29,30^.

A significant amount of work has been done so far to develop cotton cultivars that can tolerate stress. In this pursuit, plant breeders often depend on the genetic variability present in the available germplasm to select desirable traits^31^. To improve breeding programs aimed at producing salt-tolerant cotton genotypes, it is crucial to have current knowledge on genetic variability and heritability^31^. In this study, Line × Tester approach was used, where four lines and four testers were crossed, resulting in the production of 16 F_1_ hybrids. The success of improving crop plants genetically is dependent on the level of heritability of traits that are economically valuable^32^. Traits that exhibit high heritability and genetic advance are more likely to be transmitted to the next generation in greater proportions. When there is both a high h^2^b and high GAM, this can lead to genetic gains through the selection process^33^. In this study, traits such as NBP, BW, LP, SCY, K^+^/Na^+^, CAT, SOD, POD, TSP, Car, FL and FS demonstrated moderate to high heritability and GAM, suggesting the presence of additive gene action. These traits can be useful for early-stage selection of genotypes, which can then be utilized in breeding programs focused on improvement^34^. Under salt stress environment, significant reduction was observed in agronomic traits such as PH, NBP, BW, SCY, and lint%. Interestingly, fiber fineness (FF) was increased under saline environments. Furthermore, the mean values for H_2_O_2_, CAT, SOD, and POD, were increased under salt stress, while the mean values for TSP, Car, Chl a and b were decreased under saline conditions^8^. In a stress breeding program, measuring chlorophyll content is crucial in identifying salt-tolerant genotypes, as higher chlorophyll contents correlate with greater salt tolerance. In our study chlorophyll contents were reduced under salt stress conditions^35^. The reduction in chlorophyll contents, leads to increased production of H_2_O_2_^18^. This creates oxidative stress due to the strong negative association between H_2_O_2_ and chlorophyll content under salt treatment^36^. One of the ways in which oxidative stress can reduce cotton yield under salt stress is by inhibiting photosynthesis. Salt stress-induced oxidative stress can damage the photosynthetic machinery, resulting in a decrease in the efficiency of photosynthesis. This, in turn, reduces the plant’s ability to produce energy and fix carbon dioxide, which are essential for growth and yield^37–39^. Sodium and potassium are essential ions that play critical roles in different biochemical processes in plants, including osmoregulation, ion homeostasis, and enzyme activation^40^. Salt stress can cause an influx of toxic ions such as sodium and chloride into the plant and decrease the potassium contents in plants, which disrupt ion homeostasis, leading to ion toxicity and osmotic stress^3^. This can impair the plant’s ability to maintain normal cellular function, resulting in reduced cotton yield and boll weight. In our study, the genotypes FBS-FALCON × Ghauri-3, FBS-SMART × JSQ-White Hold, FBS-SMART × Barani-222, FBS-SMART × Ghauri-3, FBG-222 × JSQ-White Hold, FBG-222 × Ghauri-3, Barani-333 × JSQ-White Hold, and Barani-333 × Ghauri-3 revealed higher Na^+^ contents and lower values for the K^+^ and K^+^/Na^+^ and performed poor for agronomic physiological and fiber quality traits. The excess Na^+^ accumulation can also reduce water uptake by the plant, as well as it also competes with other ions, such as potassium and calcium, for binding sites on the root surface and within the plant^8^. This can reduce the uptake of other vital nutrients and lead to nutrient deficiencies, which can further reduce yield of the seed cotton and boll weight^8^. In our study, negative association of Na^+^ and H_2_O_2_ with fiber quality traits and agronomic as well as antioxidant traits was observed under salt stress conditions. It was reported that tolerant plants accumulated a lower amount of Na^+^ ions, while sensitive plants accumulated a higher amount of Na^+^ ions within their cells^41^. Salt-tolerant plants maintain their Na^+^/K^+^ ratio by reducing the uptake of Na^+^ from the roots, sequestering the excess Na^+^ present in the cytosol into the vacuole, and promoting the efflux of Na^+^ from root cells^41^.

Antioxidants play a critical role in protecting cotton plants from oxidative stress under salt stress. Antioxidants such as catalase, peroxidase, superoxide dismutase and non-enzymatic antioxidants (carotenoids, ascorbate and flavonoids) can neutralize ROS by donating electrons to them, preventing the formation of more ROS and the damage they can cause^4^. In response to salt stress, plants activate protective enzymes such as SOD, POD, and CAT, to enhance their ability to eliminate ROS^6,30^. The current study observed an increase in the activities of SOD, POD, and CAT in leaves under salt stress. The increased SOD activity in leaves may aid in scavenging oxygen-free radicals, while the elevated CAT activity may facilitate the breakdown of H_2_O_2_ into water and oxygen, thus reducing its levels. This finding is consistent with previous reports^6,8,10^ who also noted an increase in SOD, POD, and CAT activities in cotton leaves under salinity stress. The POD activity was enhanced up to 53% in resistant cultivars^37^. The enhancement of POD activity contributes to the improvement of photosynthetic activity, thereby highlighting the important role of antioxidant defense mechanisms in mitigating salt stress^37^. The enhanced activity of antioxidants such as SOD, POD, and CAT were associated with salt tolerance during fiber development^42^. Under stressful conditions, carotenoids tend to increase as their primary function is to protect against singlet oxygen^43–45^. High-yielding cultivars often exhibit elevated levels of CAT, TSP, and POD as they play an active role in regulating H_2_O_2_ levels by scavenging it to maintain optimal levels. The genotypes FBS-Falcon, Barani-333, JSQ-White Hold, Ghauri, along with crosses FBS-FALCON × JSQ-White Hold, FBG-222 × FBG-333, FBG-222 × Barani-222, and Barani-333 × FBG-333 revealed higher level of K^+^/Na^+^, K^+^, POD, SOD, CAT, TSP, Chla, Chlb, and CAR and declared as salt tolerant cultivars. The above-mentioned genotypes also showed relatively higher expression level of *Ghi-ERF-2D.6* and *Ghi-ERF-7A.6* at 15 dS/m and proved the role of ERF genes in salt tolerance in cotton. Similar findings also reported in cotton^46^, maize^47^, quinoa^48^ and wheat^49^.

## Conclusions

The genetic variability study of cotton germplasm for morphological, biochemical, and fiber quality traits under salt stress revealed that the genotypes the FBS-Falcon, Barani-333, JSQ-White Hold, Ghauri, along with crosses FBS-FALCON × JSQ-White Hold, FBG-222 × FBG-333, FBG-222 × Barani-222, and Barani-333 × FBG-333 performed optimally and exhibited salt tolerance based on their K^+^/Na^+^ ratio, K^+^ levels, and various enzyme activities such as POD, SOD, and CAT. Furthermore, these genotypes also exhibited higher levels of TSP, Chla, Chlb, and Car under salt stress, indicating their ability to survive under the effects of salt stress. These genotypes also showed relatively higher expression level of *Ghi-ERF-2D.6* and *Ghi-ERF-7A.6* at 15 dS/m. Importantly, these genotypes also demonstrated favorable fiber quality traits, making them potential candidates for the development of salt-tolerant cotton varieties with desirable fiber properties. Future studies focused on the molecular foundation of salt tolerance in these genotypes could facilitate the development of improved cotton varieties that can thrive in salt-affected regions and contribute to the sustainable production of cotton. Further research on the mechanisms underlying salt tolerance in cotton plants is warranted to develop a better grasp of the molecular foundation of salt tolerance and to facilitate the development of improved salt-tolerant cotton varieties.

### Supplementary Information

Supplementary Figure S1.

## Data Availability

The original contributions presented in the study are included in the article/Supplementary Material, further inquiries can be directed to the corresponding author/s.

## Abbreviations

PH: Plant Height (cm)
NBP: Number of bolls/plant
BW: Boll weight (g)
SCY: Seed cotton yield (g)
LP: Lint (%)
Na^+^: Sodium concentration
K^+^: Potassium concentration
K^+^/Na^+^: Potassium to sodium ratio
H_2_O_2_: Hydrogen peroxide (μmol/g)
CAT: Catalase (U/mg protein)
SOD: Super-oxidase dismutase (U/mg protein)
POD: Peroxidase (U/mg protein)
TSP: Total soluble Protein (mg/g FW)
Caro.: Carotenoid (mg/g FW)
Chl a&b: Chlorophyll contents a & b (mg/g FW)
FF: Fiber fineness (µg/in)
FL: Fiber length (mm)
FS: Fiber strength (g/tex)
