# Supplementary figures and images for: Investigation of salt tolerance in cotton germplasm by analyzing agro-physiological traits and ERF genes expression
Source: Sci Rep. 2024 May 23;14:11809. doi: 10.1038/s41598-024-60778-0 (PMC11116465; doi:10.1038/s41598-024-60778-0)

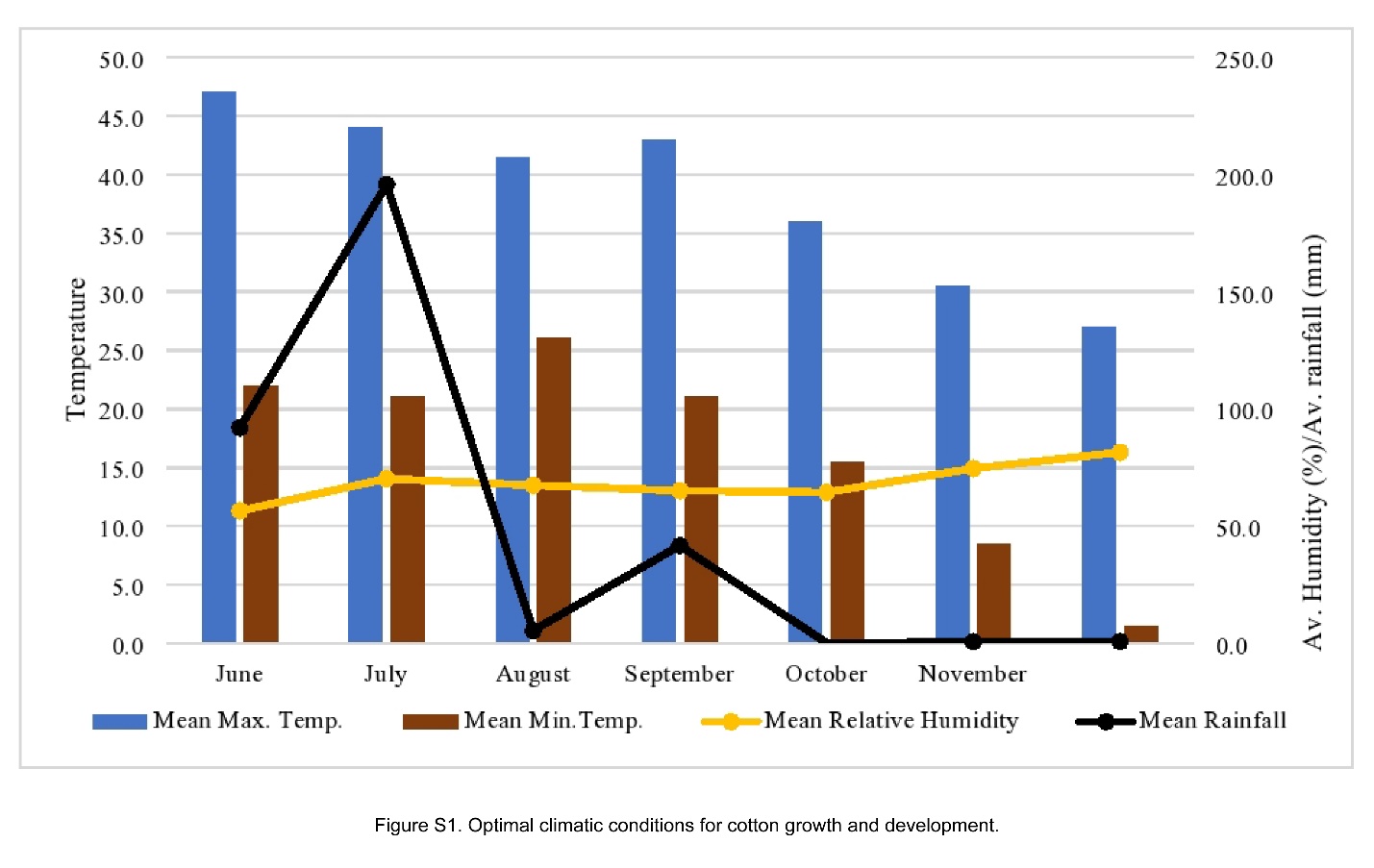


**Supplementary file**

Figure S1. Optimal climatic conditions for cotton growth and development.

Supplement: Supplementary file 1 — Supplementary Figure S1. [file 41598_2024_60778_MOESM1_ESM.docx]
